# Rad51 inhibition sensitizes breast cancer stem cells to PARP inhibitor in triple-negative breast cancer

**DOI:** 10.1186/s40880-017-0204-9

**Published:** 2017-03-30

**Authors:** Dong Wang, Ruikai Du, Suling Liu

**Affiliations:** 1 The CAS Key Laboratory of Innate Immunity and Chronic Disease, Hefei National Laboratory for Physical Sciences at the Microscale, School of Life Science and Medical Center, University of Science & Technology of China, Hefei, 230027 Anhui P. R. China; 2 Key Laboratory of Breast Cancer in Shanghai, Cancer Institute, Department of Breast Surgery, Fudan University Shanghai Cancer Center, Shanghai, 200032 P. R. China; 3 Institutes of Biomedical Sciences, Fudan University, Shanghai, 200032 P. R. China

Breast cancer susceptibility gene 1 (*BRCA1*) is a tumor suppressor gene, and its protein BRCA1 plays a role in DNA repair [1]. BRCA1 is generally expressed in the cells of mammary glands and other tissues, helping to repair damaged DNA or disrupting cells when DNA cannot be repaired. When *BRCA1* is mutated and cannot function and therefore the damaged DNA cannot be repaired on time, the risk of breast cancer will greatly increase [2]. In *BRCA1*-mutant tumors, the capability of DNA damage repair is decreased, which makes tumor cells sensitive to DNA-damaging drugs; however, high BRCA1 activity weakens the effect of these drugs [3].

Poly(ADP-ribose) polymerase (PARP) is an enzyme that detects DNA single-strand breaks and mediates DNA repair. The inhibition of PARP leads to the accumulation of DNA fragmentation, especially in patients with loss of BRCA activity, and results in cells being killed by excessive DNA damage [4]. Preclinical models show that PARP inhibition selectively targets breast cancer cells lacking functional BRCA1 [5]. Accumulating evidence shows that cancer stem cells (CSCs) are responsible for the resistance to chemotherapeutic agents [6], but it is unknown whether targeted therapy (including PARP inhibition) has the same effect. As reported in the paper entitled “RAD51 mediates resistance of cancer stem cells to PARP inhibition in triple-negative breast cancer” [7] in a recent issue of *Clinical Cancer Research*, we found that PARP inhibitor (PARPi) only effectively targets *BRCA1*-mutant bulk tumor cells and *RAD51*, a gene involved in DNA double-strand break repair [8], mediates this process, but *BRCA1*-wild-type cancer cells and *BRCA1*-mutant CSCs are resistant to PARP inhibition. *RAD51* knockdown (KD) sensitizes both *BRCA1*-mutant CSCs and *BRCA1*-wild-type cells to PARP inhibition. It suggests an effective therapeutic intervention for targeting triple-negative breast cancer (TNBC).

In this study, we tested the cytotoxic effect of olaparib (a PARPi) on four TNBC cell lines including both *BRCA1*-mutant and *BRCA1*-wild-type cell lines: SUM149, SUM159, HCC1937, and MDA-MB-231. As expected, PARPi treatment resulted in fewer viable cells in *BRCA1*-mutant cell lines (SUM149 and HCC1937) compared with those with wild-type *BRCA1* (SUM159 and MDAMB231). However, after 7 days of PARPi treatment in SUM149 and HCC1937 cells, the ALDEFLUOR-positive cells (i.e., CSCs) [9] was enriched, indicating that these cells were not affected by PARP inhibition. We thus performed an exploratory polymerase chain reaction array of 84 key DNA repair genes and found that *RAD51* has higher expression in CSCs than in bulk tumor cells in SUM149 cells. We infected SUM149 and SUM159 cells with a doxycycline-inducible *RAD51* short-hairpin RNA lentiviral system. Although not affecting CSC populations in SUM159 cells, *RAD51* KD sensitized CSCs to PARPi in SUM149 cells. These results support our hypothesis that RAD51 mediates resistance of CSCs to PARPi in *BRCA1*-mutant and *BRCA1*-wild-type breast cancer cells.

We next determined the effects of PARP inhibition and *RAD51* KD in an in vivo model of TNBC. The mice were divided into four treatment groups: vehicle control, olaparib, *RAD51* KD (doxycycline water), and combined (olaparib plus *RAD51* KD). For the mice injected with *BRCA1*-mutant SUM149 cells, *RAD51* KD and PARP inhibition significantly inhibited tumor growth in the early stage. However, when treatment was given after the tumor grew to a certain stage (2 mm in diameter), PARP inhibition was not effective, whereas the effect of *RAD51* KD was still significant. The combination of *RAD51* KD and PARPi led to a more robust inhibition of tumor growth. To assess the effects of PARP inhibition on the CSC frequency, serial dilutions of SUM149 cells (control and treated) were re-injected into mouse mammary fat pads [10]. Compared with vehicle control, *RAD51* KD alone and combined treatments reduced the CSC frequency by 70% and 90%, respectively. There is a consistent result in *BRCA1*-wild-type SUM159-injected mice. Together, these results indicate that our therapeutic regimen targets both *BRCA1*-mutant CSCs and *BRCA1*-wild-type cells (both CSC and bulk tumor populations), supporting our in vitro findings of the involvement of RAD51 in resistance to olaparib and, importantly, suggesting an effective therapeutic intervention for targeting TNBC.

In conclusion, we used in vitro models and mouse xenografts to demonstrate the importance of *RAD51* in reversing PARPi resistance of CSCs in *BRCA1*-deficient and *BRCA1*-wild-type TNBC in this study. Our findings suggest that resistance to PARPi may be overcome by targeting both CSCs and bulk tumor cells. To elucidate the clinical implications, we will further use the breast cancer patient-derived xenograft model to validate our current findings. Furthermore, by targeting RAD51, it may be possible to greatly expand the sensitivity of TNBC to PARPi, beyond those with defective BRCA1 proteins. This novel approach holds potentials for significantly improved therapies for TNBC.
